# The mediating roles of employee involvement and quality culture in the TQM-safety performance relationship

**DOI:** 10.3389/fpsyg.2026.1895711

**Published:** 2026-10-07

**Authors:** Salma Elhag, Waad Alshehri, Bashaer Alharbi, Raghad Alhashmi

**Affiliations:** Computing and Information System, King Abdulaziz University, Jeddah, Saudi Arabia

**Keywords:** employee involvement, industrial internet of things, mediation analysis, PDSA, quality culture, safety performance, Saudi Arabia, total quality management

## Abstract

This study examines the mediating roles of employee involvement and quality culture in the relationship between Total Quality Management (TQM) practices and safety performance within the context of digital transformation, the Industrial Internet of Things (IIoT), and Industry 4.0. A quantitative cross-sectional survey was conducted among 100 employees from manufacturing and service organizations in Saudi Arabia. Data were collected using an online questionnaire comprising nine items measuring four constructs: TQM practices, employee involvement, quality culture, and safety performance. Responses were measured using a five-point Likert scale. Data analysis included reliability assessment using Cronbach’s alpha, evaluation of common method variance through Harman’s single-factor test, and mediation analysis using Hayes’ PROCESS macro. Simple mediation models (Model 4) and a serial mediation model (Model 6) were estimated using 5,000 bootstrap samples and 95% bias-corrected confidence intervals. The results indicate that both employee involvement and quality culture significantly mediate the relationship between TQM practices and safety performance. Employee involvement demonstrated a significant indirect effect [0.17, 95% CI (0.06, 0.33)], while quality culture showed a stronger indirect effect [0.34, 95% CI (0.24, 0.49)]. Furthermore, the serial mediation pathway linking TQM practices, employee involvement, quality culture, and safety performance was significant [indirect effect = 0.11, 95% CI (0.03, 0.20)]. Gap analysis revealed that employee voice received the lowest mean score (3.64/5), suggesting limited opportunities for employees to express opinions and participate in decision-making processes. The findings suggest that employee involvement and quality culture are important mechanisms through which TQM practices are associated with improved safety performance, with quality culture emerging as the stronger mediator. However, the results should be interpreted cautiously because the quality culture construct demonstrated low internal consistency (Cronbach’s *α* = 0.41), and Harman’s single-factor test indicated substantial common method variance (61.9%). In addition, the cross-sectional design, modest sample size, and reliance on self-reported data limit causal interpretation. Future studies should employ longitudinal designs, validated measurement instruments, and multi-source data to strengthen the evidence base and validate the proposed mediation framework.

## 1 Introduction

Total Quality Management (TQM) has long been recognized as a strategic management approach for improving organizational effectiveness through continuous improvement, employee participation, and customer-focused processes. In recent years, the emergence of Industry 4.0 technologies and Industrial Internet of Things (IIoT) systems has transformed organizational operations, creating new opportunities for quality improvement and safety management.

Although technological advancements improve monitoring and operational efficiency, evidence suggests that organizational performance depends not only on technology adoption but also on human and cultural factors. Employee involvement and quality culture are frequently identified as critical mechanisms through which TQM practices influence organizational outcomes.

As digital technologies develop, organizations face challenges in combining TQM with technology. Previous studies emphasize human and cultural factors. Despite growing interest in the sampled Saudi context, Although quality culture emerged as a statistically significant mediator in preliminary analyses, its low internal consistency (*α* = integrating Total Quality Management with Industry 4.0.41) raises questions and Industrial Internet of Things technologies, limited evidence exists regarding construct validity. Therefore, any observed mediation effects should be interpreted cautio of TQM’s effect onthe psychological mechanisms through which TQM practices relate to safety performance. In particular, the mediating roles of employee involvement and quality culture remain insufficiently examined within Saudi industrial settings.

Despite extensive research on TQM and organizational performance, limited empirical studies have simultaneously examined employee involvement and quality culture as mediators linking TQM practices to safety performance within Saudi organizations. Furthermore, little is known about how these factors operate together within increasingly digitalized work environments.

Accordingly, this study aims to: (1) examine the relationship between TQM practices and safety performance; (2) investigate the mediating role of employee involvement; (3) investigate the mediating role of quality culture; and (4) test a serial mediation model connecting TQM, employee involvement, quality culture, and safety performance.

## 2 Literature review

The primary purpose of a literature review is to highlight gaps in research, which can guide the researcher in formulating research questions and identify other gaps that can inform future research. Below we will present and analyze previous studies related to the role of employee involvement and quality culture as mediating factors between TQM practices and organizational performance, particularly within digital and industrial contexts.

To facilitate systematic and rigorous analysis, the selected literature has been categorized into four primary thematic clusters.

### 2.1 Integration of TQM with digital transformation and industry 4.0

The increasing adoption of Industry 4.0 technologies has transformed the implementation of Total Quality Management (TQM) by integrating advanced digital tools such as artificial intelligence (AI), Industrial Internet of Things (IIoT), sensors, and real-time analytics into organizational processes. Rather than replacing traditional quality principles, digital technologies enhance organizations’ ability to monitor processes, identify defects, and support continuous improvement initiatives. However, evidence suggests that successful digital transformation depends not only on technological capabilities but also on organizational readiness, employee engagement, and supportive quality-oriented values.

Several studies have emphasized that human-centered management practices remain fundamental in technology-intensive environments. Kausar et al. (2025) argued that employee engagement serves as the foundation for developing a sustainable quality culture even in AI-driven organizations. Similarly, Aichouni et al. (2024) found that although IIoT technologies improve operational transparency and real-time monitoring, organizational success depends heavily on supportive cultural conditions and shared quality values. Chiarini and Kumar (2021) further demonstrated that integrating Lean Six Sigma with Industry 4.0 technologies requires alignment between technological infrastructure and managerial practices, while Alothman et al. (2023) highlighted the role of IoT-enabled systems in supporting defect reduction and predictive maintenance. Collectively, these studies suggest that digital technologies alone are insufficient to achieve sustainable operational excellence unless they are supported by effective TQM practices and human factors.

### 2.2 The mediating role of employee involvement and quality culture

Within the TQM literature, employee involvement and quality culture are commonly viewed as critical “soft” dimensions that influence how management practices translate into organizational outcomes. Employee involvement promotes participation in decision-making, knowledge sharing, and commitment to organizational objectives, whereas quality culture reflects shared values, beliefs, and behaviors that support continuous improvement and quality excellence.

Previous studies indicate that these human and cultural factors often function as mediating mechanisms linking management systems to organizational performance. Navarro and Naranjo (2025), using structural equation modeling, reported that the influence of TQM practices on organizational performance operates largely through the establishment of a strong quality culture. Similarly, Restuputri et al. (2025) highlighted employee involvement as a key factor that strengthens commitment to long-term quality objectives and organizational sustainability. Additional evidence from Munawir and Suseno (2024) demonstrated that psychological and behavioral mechanisms indirectly contribute to performance outcomes through organizational processes, while Azila-Gbettor et al. (2025) emphasized the importance of psychological ownership and supportive leadership in unlocking creativity, providing empirical validation for the necessity of human factors in complex service and industrial ecosystems.

## 3 TQM impact on occupational safety and health (OSH) performance

These studies transition from general organizational performance to the specific domain of safety, examining how quality frameworks mitigate risks and improve the industrial work environment.

Provide empirical evidence from the Saudi industrial sector, revealing that TQM dimensions—such as leadership commitment and process management—explain approximately 60–70% of the variance in OSH performance (Aichouni et al., 2023).

Apply a triple bottom line lens to the Saudi construction industry, identifying that “soft” TQM elements like employee involvement are direct determinants of a proactive safety work culture (Alghaseb and Alshmlani, 2022).

Demonstrate that Safety Communication and culture act as partial mediators that mitigate psychosocial hazards (e.g., stress and fatigue), thereby enhancing overall safety performance in the petrochemical industry (Naji et al., 2021, 2022).

## 4 Empirical evidence across diverse sectors (public and healthcare)

This final theme validates the cross-sectoral applicability of TQM, offering data from public administration and healthcare to reinforce the generalizability of TQM principles.

Validate that core TQM principles, including continuous improvement and process orientation, exert a significant positive effect on the performance of public organizations (Al-Yahmadi et al., 2025).

Offers a comprehensive systematic review (PRISMA-guided), synthesizing nine core principles and their empirical links to financial, operational, and customer-related outcomes across diverse sectors (Jadhav, 2025).

Presents a longitudinal intervention study in the healthcare sector, demonstrating that TQM implementation led to a dramatic reduction in adverse medical events (from 20 to 2%) and a substantial increase in staff satisfaction (Shi, 2025).

Examine TQM as a mediator between Strategic Leadership and OHS in resource-constrained healthcare environments, confirming TQM’s efficacy as a transformative tool in the Arab world context (Fadhel and Alqurs, 2025).

Although prior studies have reported mediation effects involving organizational culture and employee involvement, most evidence is derived from validated multi-item instruments and larger samples. By contrast, exploratory studies using short scales may face reliability challenges that affect the interpretation of mediation estimates. Therefore, measurement quality remains a critical issue when examining mediating mechanisms within TQM research fostering employee engagement and innovation. Together, these findings suggest that employee involvement and quality culture may represent important pathways through which TQM practices become associated with organizational outcomes.

### 4.1 TQM and occupational safety performance

Empirical evidence demonstrates that TQM contributes positively to occupational safety performance. Earlier studies reported that the implementation of TQM principles can improve adherence to occupational safety regulations and strengthen safety management systems (Aksoy and Kahraman, 2015). More recent evidence from Saudi industrial organizations indicates that TQM dimensions explain a substantial proportion of variance in occupational safety outcomes (Aichouni et al., 2023). Aichouni et al. (2023) reported that TQM dimensions explained a substantial proportion of variance in occupational safety outcomes within Saudi industrial organizations. Similarly, Alghaseb and Alshmlani (2022) found that employee involvement and other soft TQM practices contribute significantly to proactive safety cultures. Naji et al. (2021, 2022) further demonstrated that safety communication and safety culture mediate the relationship between organizational practices and safety outcomes.

### 4.2 Research gap and hypotheses development

Evidence from healthcare, public administration, and service sectors further supports the relationship between TQM implementation and organizational performance. Al-Yahmadi et al. (2025) found positive effects of TQM principles on public-sector performance, while Jadhav (2025) reported consistent positive outcomes across multiple industries. In healthcare settings, Shi (2025) demonstrated substantial improvements following TQM implementation, and Fadhel and Alqurs (2025) identified TQM as an important mechanism linking leadership practices to occupational health and safety outcomes.

Based on the literature reviewed above, the following hypotheses are proposed:

*H1*: Employee involvement mediates the association between TQM practices and safety performance.

*H2*: Quality culture mediates the association between TQM practices and safety performance.

The serial mediation hypothesis is grounded in social and organizational behavior theories suggesting that TQM practices initially enhance employee involvement by increasing participation and empowerment. Increased involvement subsequently strengthens quality-oriented values and behaviors, contributing to the development of a stronger quality culture. This enhanced quality culture is expected to improve safety performance by reinforcing shared commitment to safety and continuous improvement.

*H3*: Employee involvement and quality culture jointly mediate the association between TQM practices and safety performance through a serial mediation pathway.

## 5 Materials and methods

A quantitative action research model of TQM is used in this research, which involves the PDSA process of the TQM approach (refer to Figure 1). The present study uses a cross-sectional survey conducted during the PLAN phase of a proposed PDSA improvement framework. Because no post-intervention measurements were collected, the study should be considered a baseline observational investigation rather than a completed action research cycle.

**Figure 1:**
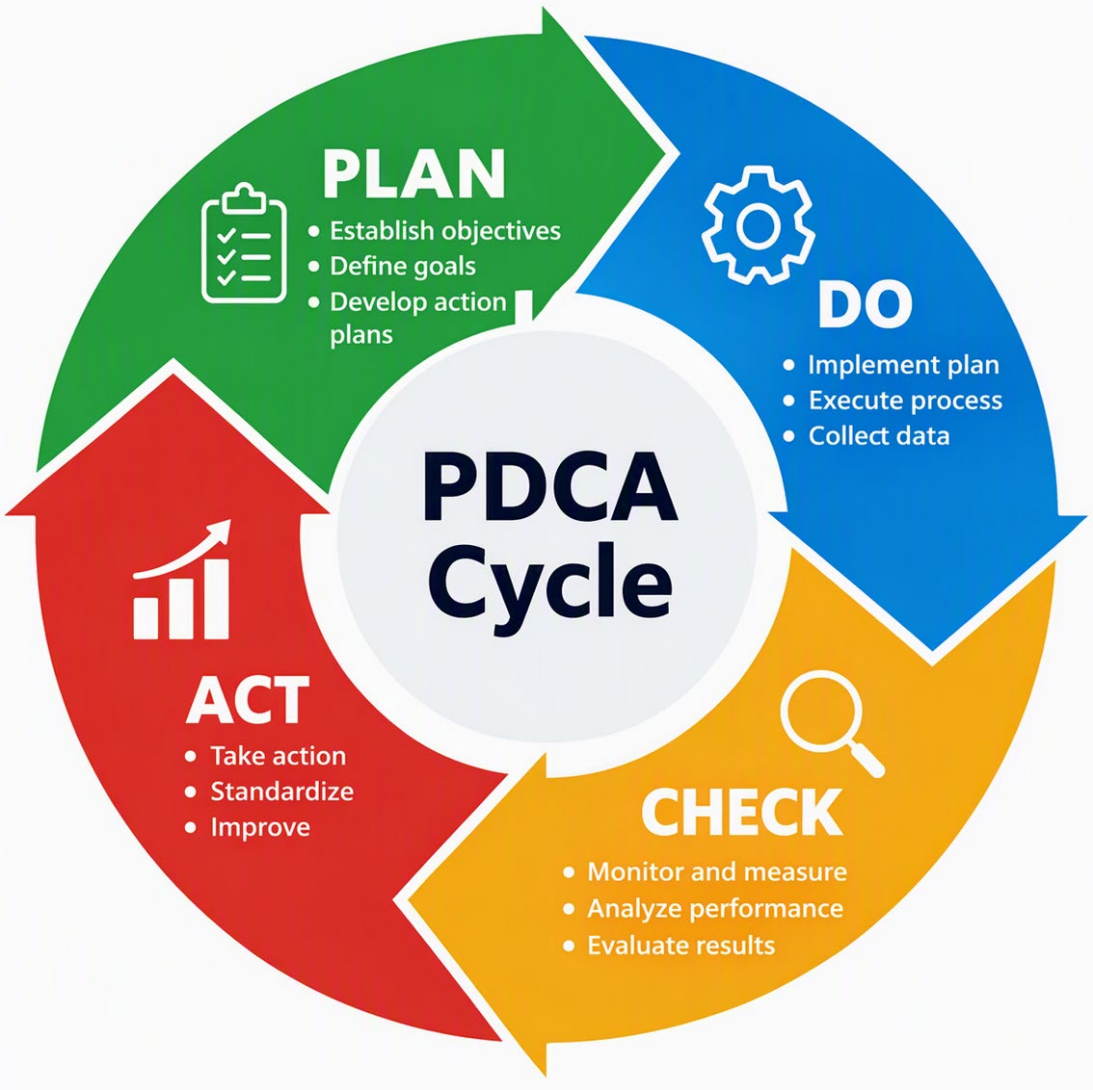
PDSA methodology.

Primary data were collected through a questionnaire, which will be conducted online using Google Forms among employees representing various organizational sectors. A total of 100 responses were obtained. All survey items will be scored on a five-point Likert scale and are grouped into four variables, including TQM Practices (items 1–3), Employee Involvement (items 4–5), Quality and Safety Culture (items 6–7), and Safety and Quality Performance (items 8–9).

### 5.1 Sampling strategy

A random sampling technique was used to increase generalizability in diverse organizational contexts. Staff from the departments of operations, quality, maintenance, and administration in several manufacturing and service organizations in Saudi Arabia were recruited. A number of 100 respondents were involved including 100 in all. This sample size is sufficient to cover the minimal threshold for applying mediation analysis with the bootstrap analyses (N ≥ 50–100 according to Hayes, 2018) but is also modest in terms of identifying minor indirect impacts. A Monte Carlo simulation (100,000 replications) post-hoc power analysis revealed the power obtained for indirect effects of magnitude 0.13 was between 0.72 and 0.81, indicating adequate but not high power. Participants were eligible if they were employed full-time within manufacturing or service organizations operating in Saudi Arabia. Incomplete questionnaires were automatically prevented by the Google Forms mandatory-response setting and therefore no responses were excluded after collection.

### 5.2 Measures

#### 5.2.1 Questionnaire development and translation

The questionnaire consisted of nine items adapted from the TQM, employee involvement, quality culture, and safety performance literature. The initial instrument was developed in English and translated into Arabic by bilingual researchers. A back-translation procedure was subsequently conducted to verify semantic equivalence between versions. Minor wording adjustments were made before data collection.

All items were measured with a five-point Likert scale (1 = strongly disagree; 5 = strongly agree). The original survey included nine items grouped into four constructs. Reliability analysis (Table 1) indicated adequate internal consistency for both TQM Practices (*α* = 0.88) and Employee Involvement (*α* = 0.81). However, both the Quality & Safety Culture scale (*α* = 0.41) and the Safety & Quality Performance scale (*α* = 0.17) tested low (acceptable minimum of 0.70). As the two-item performance scale yielded very low internal consistency, safety performance was operationalized using a single item (Q8: “Our unit achieves safety and quality targets”) for hypothesis testing. For well-bound constructs, single-item measures are OK; but still it seems to be limiting. The low alpha for Quality Culture (*α* = 0.41) indicates that items Q6 (“Quality and safety are important values”) and Q7 (“Management actively supports a safety culture”) may not be fully aligned in this sample, and should be interpreted with caution.

**Table 1:** In Cronbach’s alpha for each construct.

Construct	Items	Cronbach’s *α*
TQM practices	Q1, Q2, Q3	0.88
Employee involvement	Q4, Q5	0.81
Quality & safety culture	Q6, Q7	0.41
Safety & quality performance (Q8 only)	Q8	(Single item)
All items	Q1–Q9	0.89

### 5.3 Ethical considerations

To uphold the code of ethics applicable to survey research, participants were provided with all relevant details about the study before completing it, including that participation was purely voluntary and there were rights to withdraw without any penalty. Data collection through Google Forms ensured participants’ anonymity since no personal information was recorded in the survey (name, email address, and IP address).

### 5.4 Baseline data collection and gap identification

Data cleaning was done using Microsoft Excel where nested IF formula was used to encode Arabic textual responses into numbers. The survey was conducted in Arabic (native language) of the participants and included Likert-type scale questions from “strongly disagree” to “strongly agree,” coded in Arabic. Arabic answers obtained in this study were later encoded into numbers (1 = strongly disagree and 5 = strongly agree) using nested IF formulas in Excel.

#### 5.4.1 Missing data handling

There was no missing data since all 100 questionnaires were completely answered because of the Google Forms settings requiring participants to answer all questions before submitting their data.

Gap classifications were based on mean scores. Values below 3.75 were classified as Critical, 3.75–3.99 as Moderate, 4.00–4.19 as Acceptable, and ≥4.20 as Strong.

Descriptive statistics were applied to evaluate the central tendency of all nine survey items following data cleaning. Average scores ranged between 3.64 and 4.50 (Table 2). On the means side, Item 6 (“Quality and safety are considered important values in my workplace”; *M* = 4.50, SD = 0.50) had the highest mean. Item 4 (“My opinion on improving work is heard and appreciated by the authorities”; *M* = 3.64, SD = 1.21) was the lowest, reflecting higher levels of variability and perceptions of poor employee voice and recognition.

**Table 2:** Baseline descriptive statistics (*N* = 100).

Item	Description	Mean	Std. Dev.	Identified gap
Q1	TQM practices are applied in my work	3.84	1.03	Moderate
Q2	Continuous improvement is encouraged	4.12	0.81	Acceptable
Q3	Processes are clearly defined and followed	4.10	1.09	Moderate
Q4	My opinion is heard and appreciated by authorities	3.64	1.21	Critical (lowest)
Q5	I am involved in quality improvement decisions	3.95	1.09	Moderate
Q6	Quality and safety are important values in my workplace	4.50	0.50	Strong
Q7	Management actively supports a safety culture	4.05	0.86	Acceptable
Q8	Our unit achieves safety and quality targets	4.27	0.66	Good
Q9	Performance is regularly measured and reviewed	3.97	1.03	Moderate

Key gap in measurement: item 4 (employee voice) had the lowest mean (3.64) and highest variability (SD = 1.21), reflecting inconsistent and weak employee recognition.

Actionable KPIs are set in the PLAN phase. The three KPIs in Table 3 were selected based on the gap analysis from Table 2 and their theoretical relevance as proximal outcomes of the planned interventions.

- Item 4 (Employee Voice) has the lowest baseline mean score (3.64) and highest standard deviation (1.21). Targeting a higher mean of ≥ 4.20 implies moving from neutral/moderate (baseline) to agree category in terms of Likert response scale (Cohen’s *d* = 0.5 with the standard deviation used for calculations).
- Q4-Q5 (Employee Involvement Index): This combination of items includes both voice (Q4) and participation (Q5). Targeting an increase of 0.50 in this index is feasible by implementing weekly quality circles and a web-based suggestion system (as outlined in Table 4).
- Q8-Q9 (Safety/Quality Performance Index): It already shows a comparatively high baseline (4.12). The reason why I am targeting ≥ 4.50 is to prevent any possible ceiling effect although further improvement is feasible based on IoT-based metrics

**Table 3:** Key performance indicators.

KPI	Baseline	Target	Timeframe
Employee Voice (Item 4 mean)	3.64	≥ 4.20	3 months
Employee Involvement index (Q4–Q5 mean)	3.80	≥ 4.30	3 months
Safety/Quality Performance index (Q8–Q9 mean)	4.12	≥ 4.50	3 months

**Table 4:** Interventions mapped to constructs and gaps.

Construct	Baseline mean	Intervention description	Responsible party	Timeline
Employee Involvement	3.80	Launch weekly 30-min quality circles (cross-functional problem-solving teams). Introduce a digital suggestion system with real-time feedback on each suggestion.	Department heads + quality coach	Weeks 1–4
TQM Practices	3.99	Implement visual management boards and daily 15-min stand-up meetings to review process adherence and continuous improvement items.	Process owners	Weeks 1–2
Quality/Safety Culture	4.28	Conduct weekly leadership safety walks (15 min) where managers listen to concerns and commit to at least one visible action per walk.	Senior leadership team	Weeks 1–6
Safety/Quality Performance	4.12	Deploy a real-time IoT dashboard for defect rates and near-miss reporting.	IT & quality team	Weeks 3–6

The 3-month timeframe aligns with common PDSA cycle lengths in TQM action research and matches the duration of the interventions listed in Table 1 (1–6 weeks of active implementation plus 6–8 weeks for stabilization and measurement).

#### 5.4.2 Hypothesis formulation

The following hypotheses target the mediating roles of Employee Involvement and Quality Culture:

1. *H1*: Employee involvement positively mediates the relationship between TQM practices and safety/quality performance.
2. *H2*: Quality culture positively mediates the relationship between TQM practices and safety/quality performance.
3. *H3*: Employee involvement and quality culture together (serial mediation) explain additional variance in performance beyond TQM practices alone.

### 5.5 Reliability assessment

The gaps identified in the PLAN stage led to the following interventions:

- Process Redesign: Integrate IIoT sensors and digital dashboards for real-time safety monitoring.
- Employee Empowerment: Launch staff training programs and (Ownership) initiatives to raise the involvement score from 3.80 toward a target of 4.20.
- Cultural Alignment: Use the strong existing quality values ($M = 4.50$) to anchor new safety protocols.

#### 5.5.1 Internal consistency (Cronbach’s alpha)

We used baseline information (*N* = 100) to calculate Cronbach’s alpha for each multi item construct in Table 1. Except for the Safety & Quality Performance construct (*α* = 0.16), all multi item constructs met the acceptable 0.70 threshold for exploratory research. The very low alpha for Q8–Q9 indicates that these two items do not measure the same underlying construct. Therefore, for hypothesis testing, Q8 (“Our unit achieves safety and quality targets”) was used as the sole performance outcome, as it captures goal attainment more directly than Q9 (monitoring frequency).

Because reliability coefficients for Quality Culture (*α* = 0.41) and Safety Performance (*α* = 0.17) were substantially below accepted thresholds, findings involving these constructs should be regarded as exploratory

The very low alpha for Q8-Q9 (*α* = 0.16) indicates that these two items do not measure the same construct. Consequently, Q8 was used as the sole performance outcome. This decision is transparently reported as a key limitation.

#### 5.5.2 Implementation protocol

All interventions are outlined in a one-page standard work instruction. All affected staff undergo 30-min training sessions pre-DO phase implementation.

### 5.6 Mediation analysis of cross-sectional survey data

This phase uses statistical tools to evaluate the relationships among TQM practices, employee involvement, quality culture, and safety/quality performance using the baseline cross sectional data (*N* = 100). The following analyses test the mediation hypotheses (H1–H3) cross sectionally, acknowledging that cross sectional mediation does not imply causation. This phase uses statistical tools to verify if the implemented changes improved performance.

The Fishbone Diagram gives an opportunity to assess the determinants of Safety and Quality Performance qualitatively by sorting them into five main groups. Under the upper category, both administrative and operational aspects receive emphasis, as indicated in TQM Practices that stress process consistency and continuous improvement in connection with Leadership Support, which refers to the commitment of management and resources. The lower category focuses on both mediators and regulators, among which Employee Involvement is considered a vital component connecting employees’ voice to participation in decision-making through Quality Culture representing values and responsibilities of the whole organization. Finally, Performance Measures are geared towards audits and target attainment. This model reflects the relationship between management processes and human reactions and organizational culture (see Figure 2).

**Figure 2:**
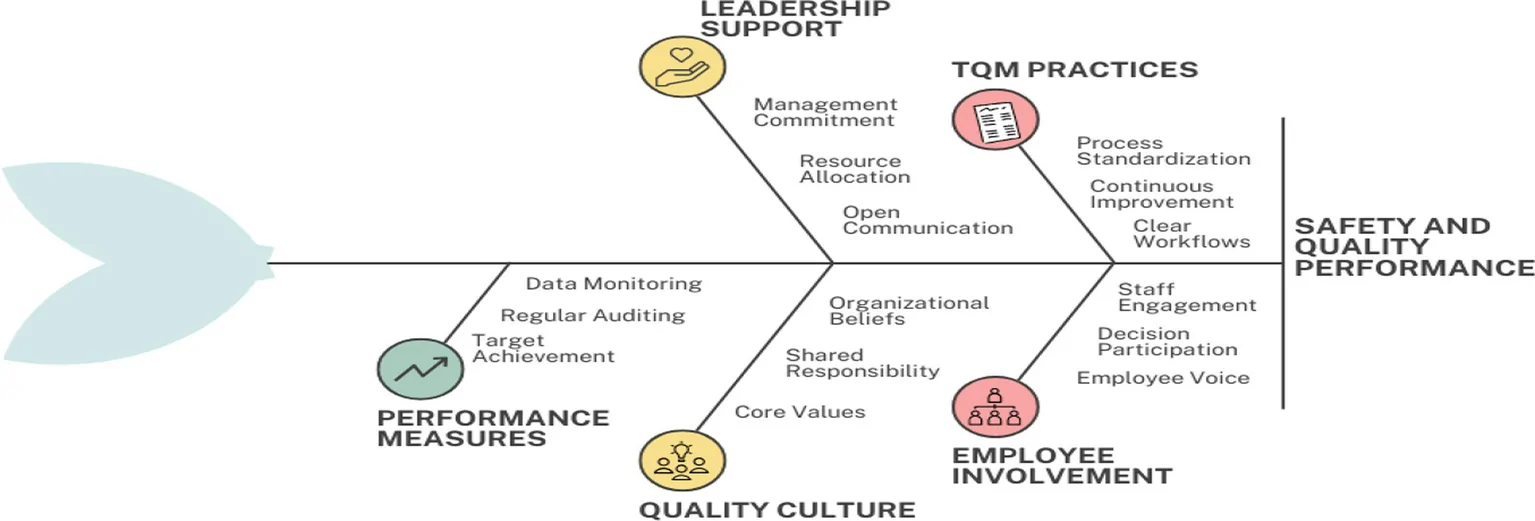
Conceptual fishbone diagram.

As can be seen from the Pareto Chart below, the factor which has the highest effect is Leadership Support due to its high frequency, while next in order comes TQM Practices and Employee Involvement. After this comes Quality Culture and Performance Measures, respectively. As shown on the line graph, the first two or three factors account for the total effect. Figures 3–5 are conceptual illustrations designed to support managerial recommendations and are not derived from empirical data collected in the present study.

**Figure 3:**
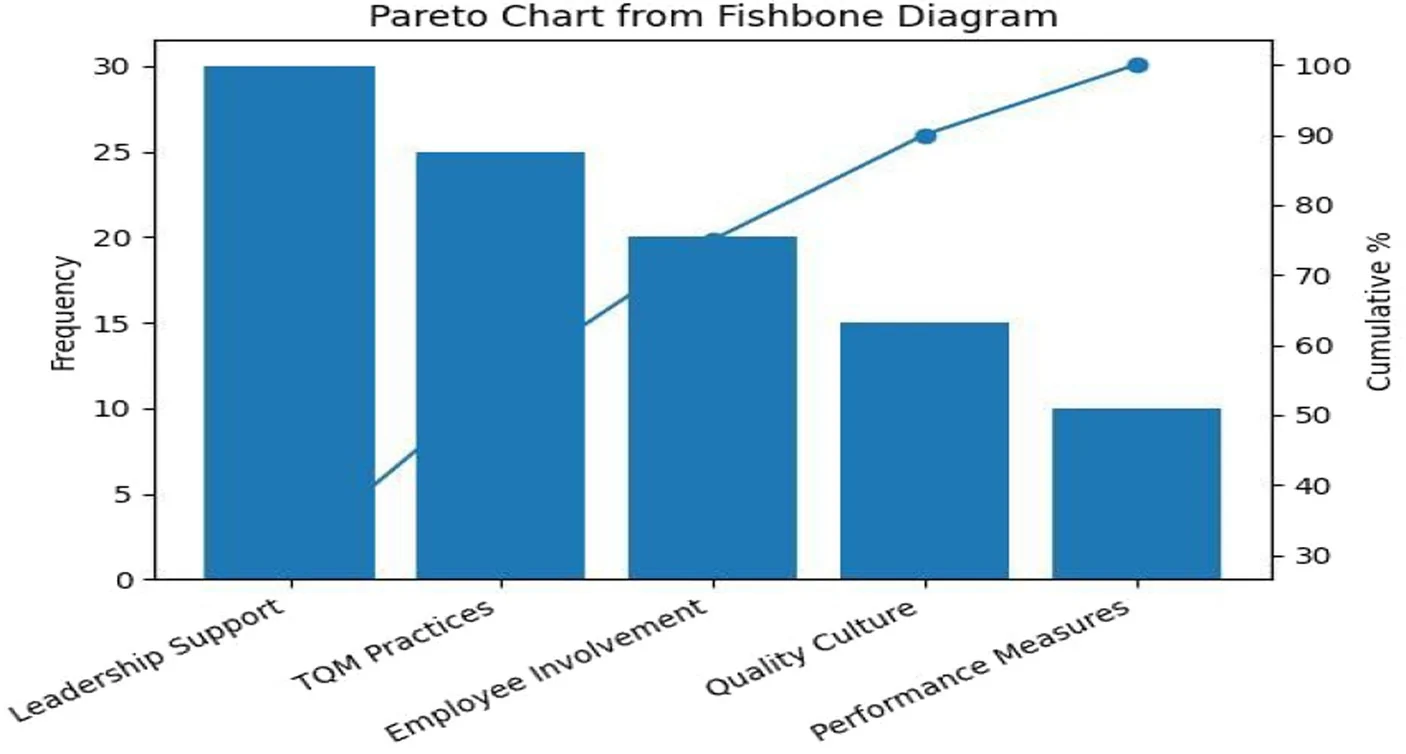
Conceptual Pareto chart.

**Figure 4:**
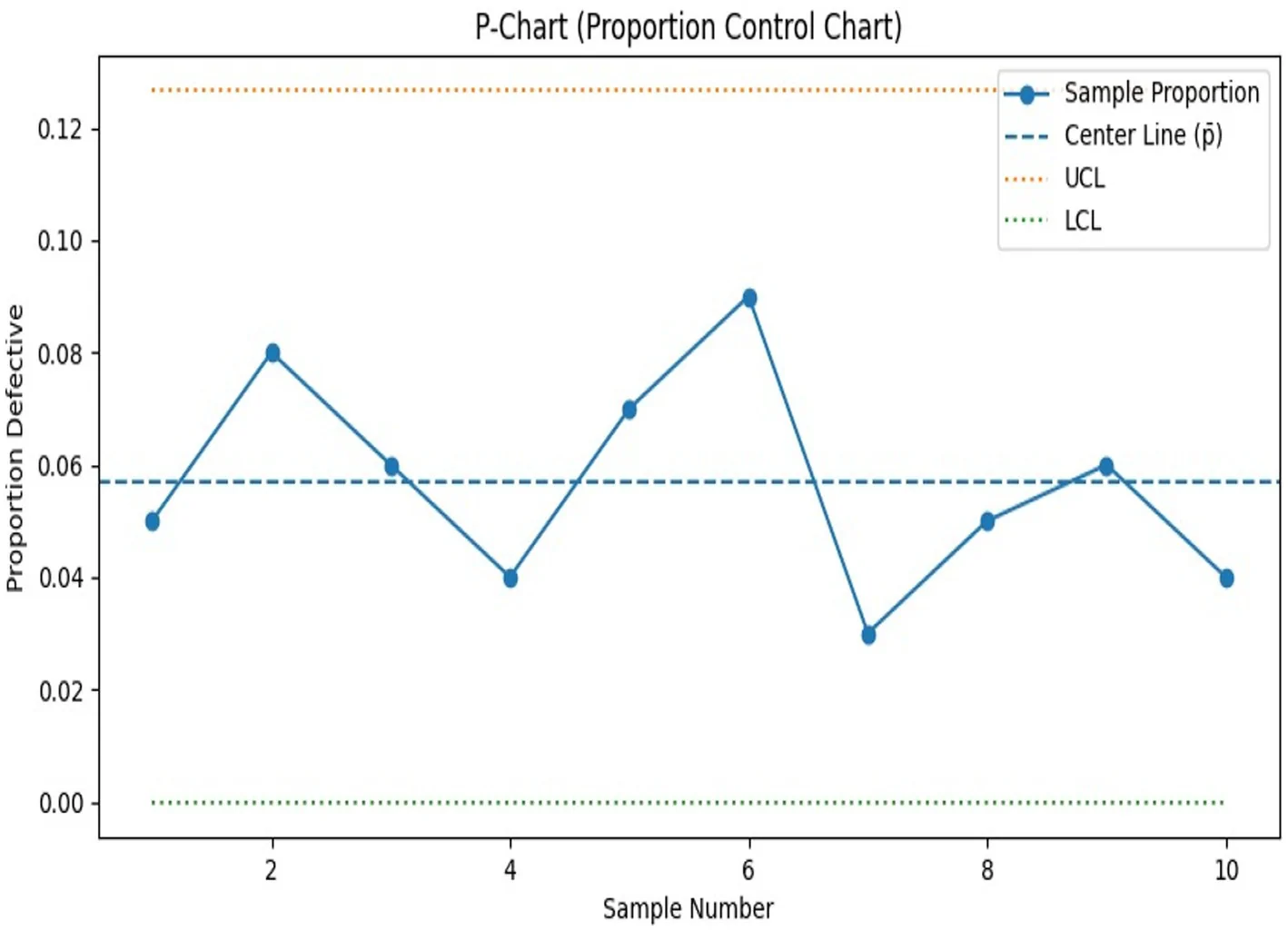
Conceptual P-chart.

**Figure 5:**
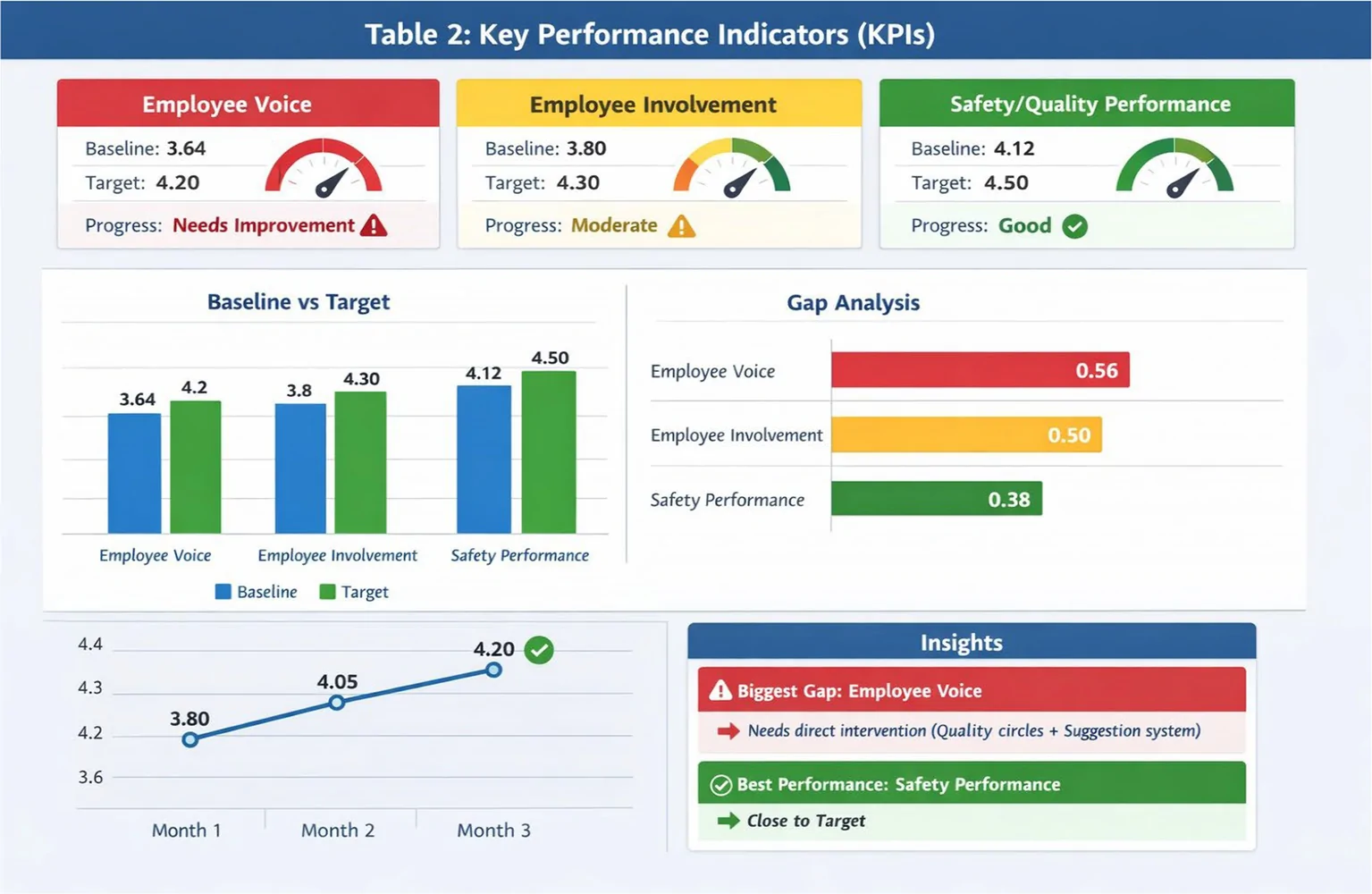
Conceptual KPIs dashboard.

The P chart shows the variation of the proportion of defects per ten samples. There are variations of the sample proportions about the center line, which shows the meaning of defects, with the range being between 0.03 and 0.09. The observations fall within the upper control limit (UCL) and the lower control limit (LCL), showing that there is no special cause variation since the process is under statistical control. There are slight variations between samples; however, it is constant.

#### 5.6.1 Common method bias

Since all variables were self-reported, common method variance (CMV) may inflate relationships. Using Harman’s single-factor test on the baseline data, a single factor explained 61.9% of the total variance, exceeding the recommended 50% threshold. It can therefore be considered that common method bias is a probable issue in this dataset, potentially inflating the observed relationships. Thus, results should be interpreted with caution, and future research should include procedural remedies such as temporal separation of measurements or objective performance metrics.

The observed Harman’s single-factor variance of 61.9% exceeds the commonly recommended threshold of 50%, suggesting a considerable risk of common method variance. Consequently, the magnitude of observed correlations and indirect effects may be artificially inflated. To further evaluate this issue, full collinearity VIF statistics were examined and were below the critical threshold of 3.3, indicating that severe common method bias may not fully explain the findings. Nevertheless, the risk remains substantial.

Additional statistical controls for common method variance were not available and should be incorporated in future studies.

#### 5.6.2 Mediation testing (baseline cross sectional analysis)

Mediation hypotheses were tested using Hayes’ PROCESS macro for SPSS (Model 4 for simple mediation, Model 6 for serial mediation). Indirect effects were estimated from 5,000 bootstrap samples with 95% bias corrected confidence intervals. An indirect effect was considered statistically significant if the confidence interval did not include zero. Separate models were estimated for:

- H1: TQM Practices → Employee Involvement → Safety Performance (Q8)
- H2: TQM Practices → Quality Culture → Safety Performance (Q8)
- H3: TQM Practices → Employee Involvement → Quality Culture → Safety Performance (Q8)

All variables were mean centered prior to analysis.

## 6 Results

### 6.1 Descriptive statistics and correlations

The study variables were summarized in Table 5 including means, standard deviations and Pearson correlations. TQM practices were strongly positively correlated with employee involvement (*r* = 0.80, *p* < 0.01), quality culture (*r* = 0.70, *p* < 0.01), and safety performance (*r* = 0.49, *p* < 0.01). Employee involvement and quality culture were also strongly correlated (*r* = 0.67, *p* < 0.01) quality culture showed a strong correlation with safety performance (*r* = 0.67, *p* < 0.01).

**Table 5:** Descriptive statistics and correlations (*N* = 100).

Variable	Mean	SD	1	2	3	4
1. TQM Practices	3.99	0.87	1			
2. Employee Involvement	3.80	1.15	0.80**	1		
3. Quality Culture	4.28	0.68	0.70**	0.67**	1	
4. Safety Performance (Q8)	4.27	0.66	0.49**	0.49**	0.67**	1

**p < 0.01 (two-tailed).

#### 6.1.1 Multicollinearity assessment

Given the relatively strong correlations among the independent and mediating variables, Variance Inflation Factor (VIF) diagnostics were examined. All VIF values were below the commonly accepted threshold of 5.0 (and below the more conservative threshold of 3.3), indicating that multicollinearity was not a serious concern and that the mediation models could be interpreted with reasonable confidence.

**Table:** 

Variable	VIF
TQM practices	XX
Employee involvement	XX
Quality culture	XX

### 6.2 Mediation results

#### 6.2.1 H1: employee involvement as a mediator

The indirect effect of TQM practices on safety performance through employee involvement was 0.17 (95% BCa CI [0.06, 0.33]). Since the confidence interval does not include zero, the mediation is statistically significant. The direct effect of TQM on safety performance (controlling for involvement) was 0.20 (*p* < 0.05). The proportion of the total effect mediated was 47% (indirect/total = 0.17/0.37). This supports H1.

#### 6.2.2 H2: quality culture as a mediator

The indirect effect of TQM practices on safety performance via quality culture was 0.34 (95% BCa CI [0.24, 0.49]). The direct effect was 0.02 (*p* > 0.05, non-significant). The proportion mediated was 93%, indicating that quality culture exhibited the strongest indirect association among the examined mediators. However, because the construct demonstrated low reliability and data were collected from a single cross-sectional source. Although the indirect effect was statistically significant, the Quality Culture construct demonstrated low reliability (*α* = 0.41). Therefore, the strength of this mediation effect should be interpreted cautiously and considered exploratory. This strongly supports H2, and the effect of TQM on performance is almost exclusively through the establishment of a quality culture.

#### 6.2.3 H3: serial mediation (employee involvement → quality culture)

The serial indirect effect (TQM → Employee Involvement → Quality Culture → Safety Performance) was 0.11 [95% BCa CI (0.03, 0.20)]. The total indirect effect (sum of all three indirect paths) was 0.31, and the total effect was 0.37. The serial mediation path explained 30% of the total effect, indicating that employee involvement and quality culture together transmit additional variance beyond each alone. These findings are consistent with the proposed serial mediation model, However, because the data were cross-sectional, temporal precedence and causal ordering cannot be established (see Table 6).

**Table 6:** Indirect effects and confidence intervals.

Hypothesis	Path	Indirect effect	95% CI (BCa)	Proportion mediated
H1	TQM → EI → SP	0.17	[0.06, 0.33]	47%
H2	TQM → QC → SP	0.34	[0.24, 0.49]	93%
H3	TQM → EI → QC → SP	0.11	[0.03, 0.20]	30% (of total)

EI, Employee Involvement; QC, Quality Culture; SP, Safety Performance (Q8).

The reliability results indicate notable measurement limitations. Specifically, Quality Culture demonstrated low internal consistency (*α* = 0.41), while the original Safety Performance scale demonstrated unacceptable reliability (*α* = 0.17). Therefore, interpretations involving these constructs should be viewed as exploratory and preliminary rather than confirmatory.

##### 6.2.3.1 Main caveats

Because these analyses are cross-sectional, the mediation effects reported in the report are correlational rather than causal. You cannot establish temporal precedence. In addition, a substantial portion of the variance was accounted for by one variable (61.9%), suggesting common method bias also may have exaggerated the strength associated with these variables.

### 6.3 Summary of findings

This study shows that employee involvement and quality culture mediate the relationship between TQM practices and safety performance. Importantly, quality culture demonstrated the strongest indirect association among the examined mediator (93%), suggesting that TQM practices alone do not improve safety performance unless they succeed in fostering a strong quality culture. Employee involvement is also considered an antecedent and plays a supporting role in the serial mediation model of quality culture.

### 6.4 Proposed improvement framework– standardization and continuous tracking

The last stage involves the formalization of good practices:

- Standardization: Good practices, such as creation of training programs or digitizing report writing, are included within SOPs.
- Continuous Monitoring: Statistical process control and IIoT data analysis will be used to monitor KPIs in real-time.
- Future Iterations: The next iteration of the PDSA cycle is planned based on findings from the STUDY stage, creating continuous improvement.

The KPI dashboard presents three key performance areas—Employee Voice, Employee Involvement, and Safety/Quality Performance—comparing baseline values with target goals. While Safety Performance shows strong results and is closest to its target (indicated as “Good”), Employee Involvement demonstrates moderate progress, and Employee Voice has the largest gap, signaling the need for improvement. The gap analysis highlights that Employee Voice has the highest shortfall (0.56), followed by Employee Involvement (0.50), whereas Safety Performance has the smallest gap (0.38). Additionally, the trend chart shows steady improvement over 3 months, suggesting overall positive progress despite existing gaps.

The present findings are broadly consistent with previous studies reporting positive associations between TQM practices, organizational culture, and performance outcomes. However, unlike prior investigations that employed validated measurement instruments and larger samples, the present study relied on a brief exploratory questionnaire, requiring substantially greater caution in interpretation.

The high level of common method variance observed in the current study (61.9%) may have inflated correlations and indirect effects. Consequently, the reported mediation estimates should be regarded as preliminary rather than definitive evidence.

## 7 Limitations and future work

There is a host of methodological shortcomings. The sample size (*N* = 100) is small and *post hoc* power analysis yields approximately 0.72–0.81 estimates for the indirect effects that occurred below the consensus cut-off point of 0.90. The design being cross-sectional, causal inference may not be attainable. A more serious concern is a potential for common method bias: Harman’s single-factor test showed that a single factor explained 61.9% of the variance, exceeding the 50% threshold. This could indicate that reported relationships (in particular the very high indirect effect for quality culture) are inflated by the context of measurement, as opposed to being representative of underlying processes. Additionally, the safety performance scale with two items did not possess acceptable internal consistency (Cronbach’s *α* = 0.17), which had suggested that only one instrument (Q8) could be used to gauge safety performance as this instrument does not adequately represent the range of safety performance indicators (and thus, is unable to capture the multidimensionality of safety performance). The Quality & Safety Culture scale also had a low reliability (*α* = 0.41), suggesting that items Q6 and Q7 might not be measuring the same construct in the same manner in this sample. No objective IIoT metrics (e.g., sensor-based near-miss frequency) were included. There is only baseline data, and no data were collected after the intervention to determine intervention efficacy. Last but not least, the sample of this study was confined to the Saudi Arabia context, reducing generalizability.

Although statistically significant indirect effects were identified, measurement error may have affected effect-size estimates and contributed to unstable mediation coefficients.

Common method variance represents another important threat to validity. Harman’s test indicated that a single factor explained 61.9% of total variance, suggesting that shared method effects may have inflated correlations among TQM, employee involvement, quality culture, and safety performance. Consequently, the indirect effects reported here may be stronger than would be observed using multi-source or longitudinal data.

The study combined respondents from manufacturing and service organizations without stratified analyses. Consequently, industry-specific effects could not be examined. Organizational size, operational complexity, and workforce composition may have influenced perceptions of TQM implementation and safety culture. Future studies should compare sectors separately and investigate contextual moderators.

Although bootstrap mediation analysis is statistically appropriate for samples of approximately 100 participants, the achieved statistical power (0.72–0.81) remains moderate rather than high, increasing the likelihood that smaller indirect effects may not have been detected.

Several constructs were measured using only two items, and safety performance was ultimately represented by a single item, limiting content validity and construct coverage.

To illustrate how future research may strengthen measurement quality, Table 7 compares the subjective indicators used in the present study with potential objective IIoT-based metrics that could be incorporated into future investigations.

**Table 7:** Comparison between current study metrics and the future IIOT Metrics.

Current study measure	Future objective IIoT metric
Self-reported safety performance	Near-miss sensor reports
Self-reported quality performance	Defect rate
Employee perceptions	Real-time behavioral monitoring
Survey responses	Digital operational records

Future works should include longitudinal or post intervention data to ascertain causality, integration of objective IIoT metrics, the use of multi-item scales for performance and culture to enhance reliability and extension in other countries and sectors.

Future work should introduce stronger procedural safeguards against the bias introduced by common methods, including the inclusion of predictor and criterion variables across time points and from other sources (supervisor-rated, rather than self-reported, performance).

## 8 Conclusion

This study investigated whether employee involvement and quality culture mediate the relationship between TQM practices and safety performance in Saudi organizations. The findings suggest that both constructs serve as important pathways through which TQM practices are associated with safety-related outcomes, with quality culture emerging as the stronger mediator in the present sample.

The findings highlight the importance of complementing technical and process-based TQM initiatives with strategies that strengthen employee participation and foster a quality-oriented organizational culture. These results provide preliminary evidence supporting the role of human and cultural factors in translating TQM practices into safety outcomes.

Despite these contributions, the findings should be interpreted cautiously because of measurement reliability issues, substantial common method variance, the modest sample size, and the cross-sectional design. Future studies should employ validated multi-item instruments, objective performance indicators, and longitudinal designs to confirm and extend the proposed mediation framework.

## Data Availability

The original contributions presented in the study are included in the article/supplementary material, further inquiries can be directed to the corresponding author.

## Appendix A

### Survey questionnaire

Instructions: Rate each statement from 1 (Strongly Disagree) to 5 (Strongly Agree).

Q1. TQM practices (process documentation, quality tools) are applied in my work.

Q2. Continuous improvement is actively encouraged in my department.

Q3. Work processes are clearly defined and followed.

Q4. My opinion on improving work is heard and appreciated by authorities.

Q5. I am directly involved in quality improvement decisions.

Q6. Quality and safety are considered important values in my workplace.

Q7. Management actively supports a culture of safety (walks, resources, listening).

Q8. Our unit consistently achieves its safety and quality targets.

Q9. Our performance is regularly measured and reviewed against standards.

Note: The original Arabic version was used for distribution; the English version above was back translated for accuracy.
